# Case Report: Complete response with combination oncolytic virus immunotherapy in a patient with stage IV renal cell carcinoma—a promising innovative approach

**DOI:** 10.3389/fonc.2025.1631155

**Published:** 2025-08-07

**Authors:** Benjamin Gesundheit, Chaim Brauns, Thomas J. Vogl, Alexander Muckenhuber, Christine Weisslein, Harald Schmoll, Ronald Ellis, Yehudit Posen, Jayadeepa Srinivas Raju

**Affiliations:** ^1^ Research and Development, RapoYerape Ltd., Jerusalem, Israel; ^2^ Department of Radiology and Nuclear Medicine, Frankfurt University Hospital, Frankfurt am Main, Germany; ^3^ Institute of Pathology, Technical University of Munich, Munich, Germany

**Keywords:** renal cell carcinoma, oncolytic virotherapy, intratumoral, immunotherapy, abscopal effect

## Abstract

Metastatic renal cell carcinoma stage IV (RCC-IV) remains a therapeutic challenge, with an overall 5-year survival rate of 12%. Conventional chemotherapy and radiotherapy have shown relatively low efficacy in reducing morbidity and mortality, whereas innovative immunotherapies have demonstrated promising clinical results with fewer adverse events (AEs). Oncolytic virus (OV) immunotherapy has produced remarkable therapeutic effects in many solid tumors, including refractory and end-stage tumors, with intratumoral (IT) injection (IT-OV) suggested to enhance both efficacy and tolerability. We report the clinical course of a patient with RCC-IV who was treated over a period of 3 years with multiple IT injections of various OVs and another immunotherapeutic agent. A complete response—confirmed through periodic radiological surveillance and biopsies—was achieved without any serious side effects, hospital admissions, or surgical interventions throughout the entire treatment course. To our knowledge, this is the first documented case of complete remission of RCC-IV mediated by IT-OV therapy. The rationale and potential of IT-OV therapy as an innovative approach for treating RCC-IV are discussed.

## 1 Introduction

Treatment of renal cell carcinoma (RCC) remains a clinical challenge. While early-stage RCC can be cured in 70%–80% of patients, the 5-year survival rate for stage IV RCC (RCC-IV) remains 12% (1–4). This poor prognosis persists despite the availability of various treatment options, including partial/total nephrectomy, immunotherapies, and tumor ablation via cryotherapy or radiofrequency. Current management primarily focuses on prolonging survival while maintaining a good quality of life (QoL). Therefore, novel therapeutic modalities are needed (5).

Various oncolytic viruses (OVs) have demonstrated positive therapeutic effects and an acceptable safety profile (6) against several solid tumor types, including refractory and incurable cancers. The therapeutic effect of intratumorally injected OVs (IT-OV) is driven by a high localized concentration of the virus, leading to rapid oncolysis while sparing normal cells (7). The IT-OV-damaged or lysed circulating tumor cells (CTC) subsequently activate the endogenous immune system (8), thereby “priming” immune cells to recognize and eliminate residual tumor cells. This results in an abscopal effect, with regression of both the IT-OV-targeted tumor and peripheral metastases (9, 10). Combining two or more OVs can enhance the oncolytic effect (11, 12), and genetically engineered OVs have demonstrated even greater therapeutic potential (13). IT-OV is significantly more effective than systemically administered OVs, as the high localized concentration within the tumor tissue induces potent local oncolysis and is associated with fewer adverse events (AEs) compared to systemic OV treatment (14, 15). Moreover, IT-OVs directly overcome the immunological defense mechanisms of the tumor microenvironment, making them more effective than systematically delivered OVs.

RCC-IV patients are considered good candidates for minimally invasive IT-OV therapy, as their treatment is typically palliative. In addition, RCC tumors are often readily accessible via ultrasound (US) or CT, which can be used to guide IT-OV injections. Furthermore, OV immunotherapy has demonstrated clinical success in the treatment of urological cancers (16). Based on this rationale, the newly diagnosed RCC-IV patient described below was treated with IT-OV.

## 2 Case presentation

A previously healthy 55-year-old man presenting with sudden-onset abdominal and flank pain (February 2020) was diagnosed with an 11 cm × 9 cm × 9 cm renal mass. Initial ultrasound (US) assessment revealed near-total displacement of the left renal architecture and rupture of Gerota’s fascia along the lower pole, likely causing the clinical symptoms. Radiological evaluation was consistent with RCC-IV, and biopsy confirmed papillary RCC type 1, WHO-/ISUP-grade 1 (17). The patient was offered standard conventional treatment, however, aware of its limited prospects for success and the potential for significant AEs with poor QoL, he opted for experimental treatment instead.

The experimental personalized IT-OV treatment, offered on a compassionate-use basis (*Individueller Heilversuch*), was discussed in detail with the patient. After providing signed informed consent, the patient received CT-guided IT-OV injections, which included Newcastle disease virus (NDV), reovirus 3 (REO3) virus, vaccinia virus, and/or parvovirus. A total of 23 IT-OVs were administered over a 35-month period. Treatment intervals were adjusted based on clinical course and radiological response OVs 1–6 every 2 weeks, OVs 7–10 every 4 weeks, OVs 11–18 every 2 months, and OVs 19–23 every 3 months (**Table 1**). OVs were titrated to dosages based on patient tolerance: NDV = 8.5 × 10^8^–4.1 × 10^10^, REO3 = 9.4 × 10^8^–1.0 × 10^10^, and vaccinia = 1.0 × 10^7^–1.0 × 10^8^. A rigorous surveillance strategy, aligned with previously published recommendations (18), was adopted. This included clinical assessments, laboratory investigations, and imaging studies. The follow-up schedule was individualized and guided by the patient’s evolving clinical status.

**Table 1:** Oncolytic virus treatment summary.

Treatment sessions	The mean interval between treatments	NDV	REO3	Vaccinia	Parvovirus
	Days (range)	PFU	PFU	PFU	PFU
1–6	16 (15–19)	4.1 × 10^9^	1.0 × 10^10^	1.4 × 10^7^	–
7–10	20 (17–24)	8.5 × 10^8^	1.0 × 10^9^	1.0 × 10^7^	–
11–17	60 (26–113)	1.0 × 10^9^	9.4 × 10^8^	2.3 × 10^7^	–
18–23	77 (35–140)	1.0 × 10^10^	6.7 × 10^9^	1.0 × 10^8^	1.0 × 10^8^

NDV, Newcastle disease virus; PFU, plaque-forming unit; REO, reovirus.

**Figure 1:**
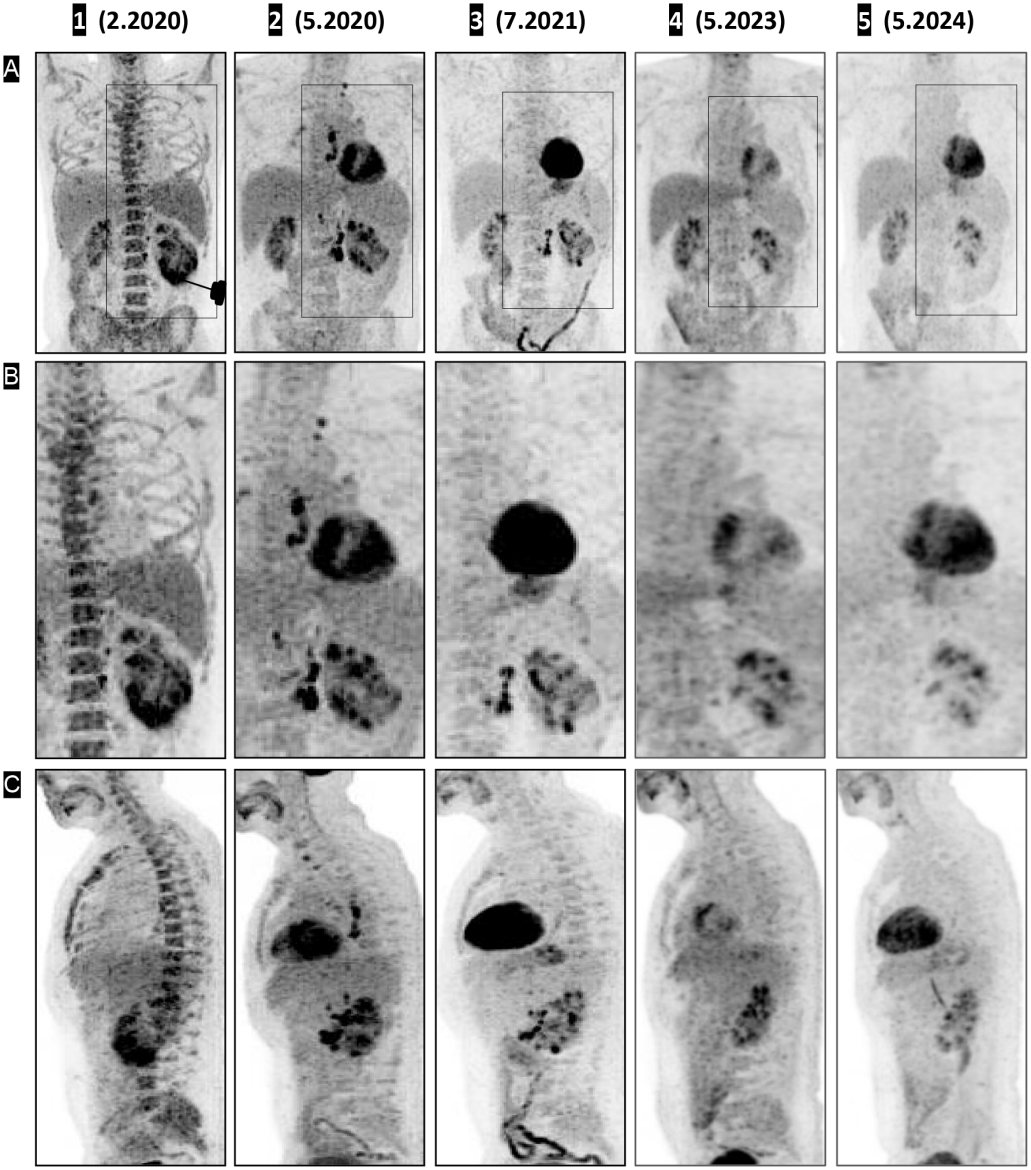
CT, PET, and fused PET-CT images demonstrating shrinkage of the renal mass (coronal view) and urine cast of necrotic tumor tissue. The treatment response timeline of the left renal primary lesion is shown in coronal view panels as contrast-enhanced CT **(A** 1–5**)**, fused PET **(B** 1–5**)**, unfused PET CT **(C** 1–5**)**, and 3D volume-rendered images using angio **(D** 1–5**)** and soft-tissue **(E** 1–5**)** presets with digital subtraction of background anatomy. Contrast CT on **(A** 1**)**, fused PET CT on **(B** 1**)**, and unfused PET on **(C** 1**)** shows a large left renal mass with intense pathological uptake, followed by a decrease in size on **(A** 2, 3**)** and a small residual scar on **(A** 4**)**. On **(A** 5**)**, there is interval development of a possible focus of dystrophic calcification. **(B** 2–**C** 2, B 3–**C** 3**)** Significant decrease in uptake, with complete resolution of pathological uptake on **(B** 5–**C** 5). The 3D volume-rendered images in the angio preset **(D** 1**)** and soft-tissue preset **(E** 1**)** show PET-correlated intense uptake in the lower pole, appearing as a thickened lower pole cortex. **(D** 2–**E** 2**)** Decreased uptake with a central deficit corresponding to necrotic debris; **(D** 3–**E** 3, **D** 4–**E** 4, **D** 5–**E** 5**)** Restored physiologic uptake.

**Figure 2:**
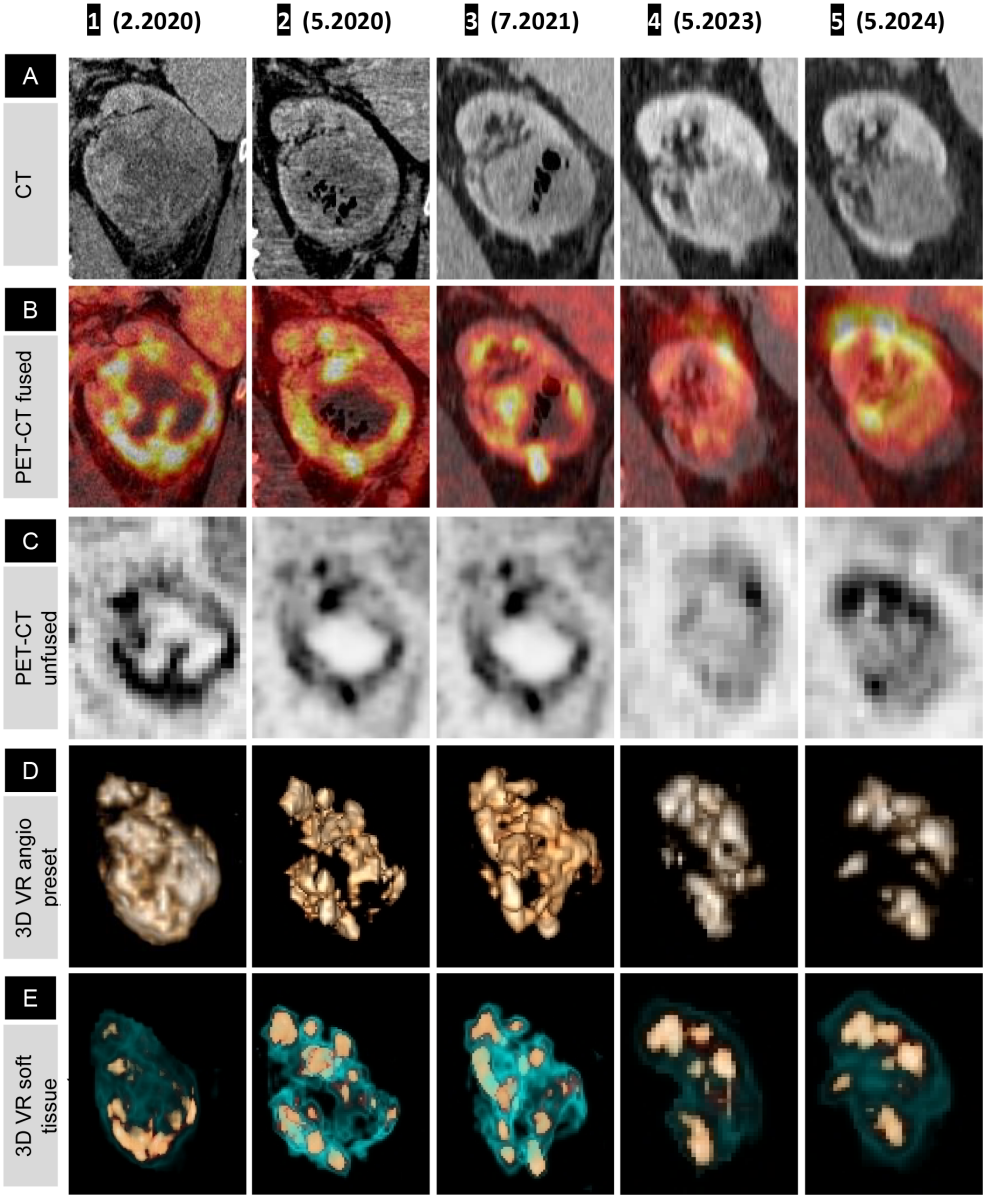
Fused PET-CT timeline and graphs showing shrinkage of renal cell carcinoma, left adrenal, and metastatic lymph nodes (coronal view). Rainbow spectrum fused PET-CT image panels demonstrating lesion-wise treatment response over the course of treatment, presented anatomically from top to bottom: left supraclavicular LN **(A)**, paraesophageal LN **(B)**, right retrocrural LN **(C)**, left retroperitoneal LN **(D)**, left adrenal **(E)**, and left kidney **(F)**. **(A** 1–**F** 1**)** Pathological uptake (red) with a faint halo of immune-mediated inflammatory flare (yellow). **(F** 2**)** Oncolytic response with a necrotic, ametabolic central core and subsequent normalization of the renal outline and function on **(F** 3–5**)**; note the phase misregistration artifact in **(F** 4**)**. **(E** 1**)** Pathological uptake in the left adrenal gland, with decreasing uptake in **(E** 2, 3**)** as a partial response to treatment, and in **(E** 4, 5**)**, a normal-sized adrenal with no uptake, confirming NED. **(A** 1–**D** 1**)** Hyperimmune activation of all four LN stations, most intense at the regional left retroperitoneal LN **(D** 1**)**. **(A** 2–**D** 2**)** Transient metabolic flare phenomenon, demonstrated by increased size and metabolic uptake with smudged margins corresponding to an inflammatory halo. This is followed by a gradual decrease in uptake on **(A** 3–**C** 3**)**, with complete interval resolution of pathological uptake in the distant LN **(A** 4–**C** 4**)** and minimal residual uptake in the left retroperitoneal LN **(D** 4) = **Figure 3B** (4)), which resolves completely on **(D** 5**)**. Graphical representations of lesion volumetry and metabolic uptake, expressed as maximum SUV, across the milestone times are presented on the right-hand side for correlation.

**Figure 3:**
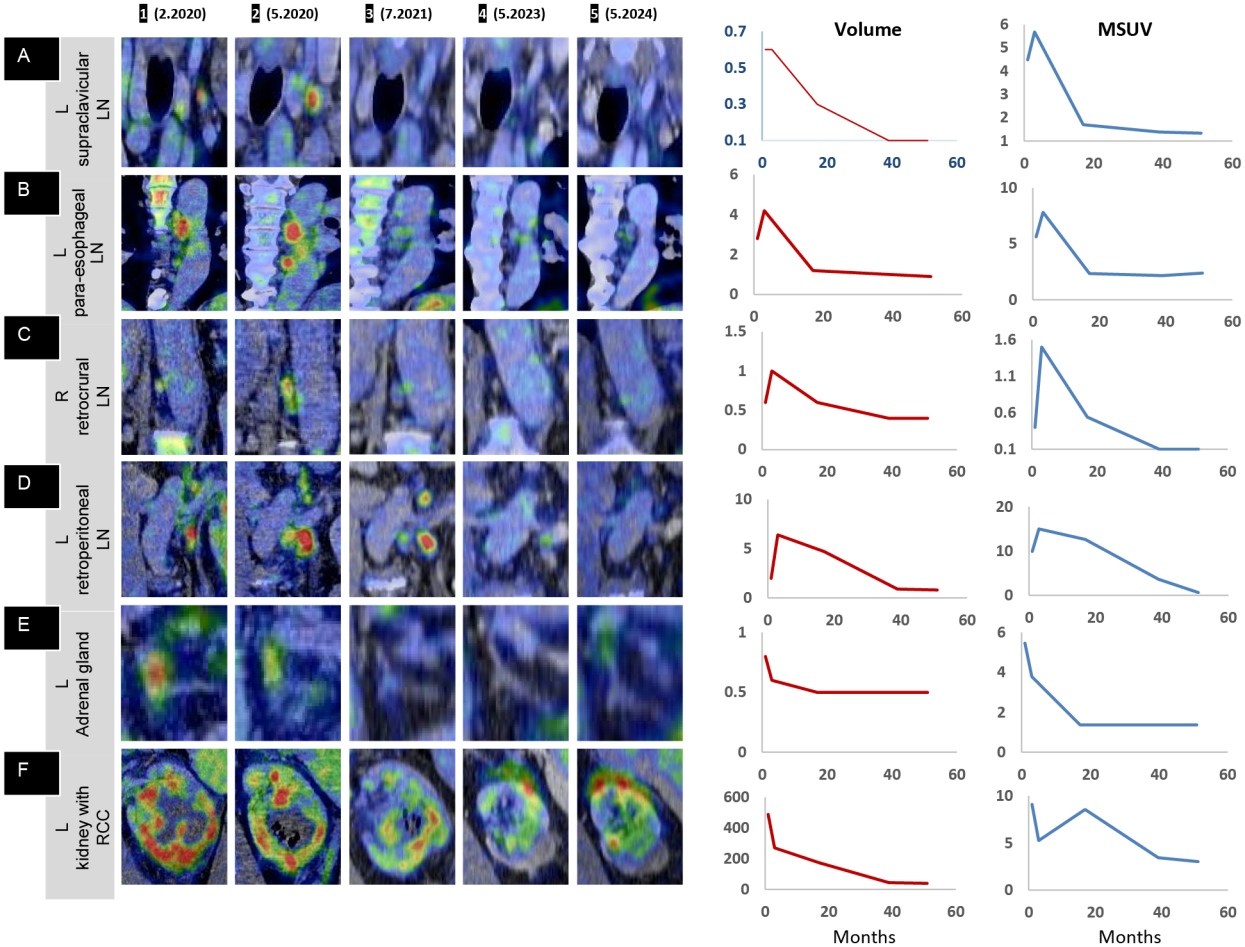
PET overview: shrinkage of renal cell carcinoma following intratumoral oncolytic virus immunotherapy. **(A, B)** Coronal and **(C)** sagittal 3D maximum intensity projection (MIP) images demonstrating the route of lymphatic spread and the treatment response in the whole body **(A** 1–5**)**, a magnified view of the abnormal uptake in the left upper-mid torso depicting the lymphatic response **(B** 1–5**)**, and sagittal midline MIP images **(C** 1–5**)** showing the initial immune activation in the axial skeleton. **(A** 1, **B** 1**)** Intense uptake in the lower pole of the left kidney (orange outline on **(B** 1**)**. **(A** 2, **B** 2**)** Immune activation uptake in the pathological left retroperitoneal LN, extending up to the left supraclavicular LN, with increased size, number, and uptake in LNs along the route of spread (in red outline). **(A** 3, **B** 3**)** Resolution of uptake at distant sites, with lower persistent uptake in the left retroperitoneal region. **(A** 4, **B** 4**)** Near-complete resolution in the left retroperitoneal region, with a single LN showing minimal uptake (red outline). **(A** 5, **B** 5**)** Complete resolution with no evidence of disease. **(C** 1**)** Transient intense uptake in the axial skeleton (vertebrae and sternum); **(C** 2–5**)** normal baseline levels, confirming a one-time initial immune activation at the precursor level (bone marrow).

Whole-body PET with FDG and CT with intravenous contrast (**Figures 1A** (1), (**B**) (1)), performed 7 days after the first IT-OV treatment revealed an 11-cm heterogeneously enhancing mass in the lower-mid pole of the left kidney, with a significant mass effect on the renal collecting system. The renal architecture was distorted, the adjoining calyces were compressed, and the lower pole of Gerota’s fascia was disrupted. The mass demonstrated pathological intense uptake, distinct from physiological renal uptake. The left adrenal gland was thickened and demonstrated increased uptake. Metastatic nodal uptake with enlarged sizes was observed in the left retroperitoneal, right retroperitoneal, paraesophageal, and supraclavicular lymph nodes (LN), confirming TNM stage IV (T4N1M1) (**Figures 2A**(1)–**D** (1), **3B** (1)). Diffuse, mildly increased uptake in the axial skeleton—attributed to immune-mediated stimulation of bone marrow at the precursor level—was noted (**Figure 3C** (1)). No focal bone uptake suggestive of osseous metastases was found.

### 2.1 Left renal primary

Three months after initiation of IT-OV treatment, a 45% decrease in the renal mass volume was observed (**Figure 1A** (2)), along with a 42% decrease in metabolic activity, as assessed by maximum standardized uptake value (MSUV), and the evolution of a central ametabolic necrotic area or oncolytic cavity (**Figures 1B** (2), **C** (2)). Additional observations included the restored intactness of the Gerota’s fascia along the lower pole and small intracavitary air loculi from the IT-OV administration, which resolved over time (**Figure 1A** (2, 3)). There was also relieved compression on the excreting interpolar calyces in the upper pole which assumed physiological excretory function (**Figure 1B** (2)). Subsequent assessments demonstrated a continued steady reduction in tumor size, reaching a 90% reduction from baseline, along with a 70% reduction in pathological metabolic uptake, accompanied by a functional 18F-fluoro-deoxyglucose (FDG) excretion via the renal tubules and preserved function following IT-OV treatment. The upper pole and the interpolar regions of the left kidney remained anatomically and functionally viable. The size, contour, and function of the left kidney were fully restored, with residual ametabolic scar tissue in the lower pole (**Figures 1**, **2**).

### 2.2 Left adrenal gland

On the first PET-CT, the left adrenal gland demonstrated increased uptake and mild thickening, which steadily declined with continued treatment, eventually returning to normal anatomical configuration and uptake (**Figure 2D**).

### 2.3 LN involvement

The left retroperitoneal LN adjacent to the kidney exhibited the highest immunogenic sensitivity, with increased metabolic uptake, most pronounced in the immediate anatomical vicinity of the primary tumor mass (**Figure 3A** (2), **B**(2), **Figures 2A–D**). A similar, though milder, pattern was observed in the retrocrural LN, likely due to its more distant location from the site of OV injection and its distinct blood supply. Similarly, the paraesophageal and left supraclavicular LNs exhibited initial intense immune-mediated flare-ups following IT-OV treatment, followed by a gradual reduction in size until they returned to a nonpathological state. After 35 months, no metastatic spread was observed, and even the highly reactive left retroperitoneal LN had returned to a subcentimeter nonpathological state, with a 70% reduction in uptake from baseline (**Figure 3A** (4), **B**(4), **Figure 2D** (4)). No intermittent changes with respect to kidney volume or metabolic uptake were observed. Lesion volumetry, correlated with metabolic uptake across treatment milestones, demonstrated a consistent graphical relationship across all time points (**Figure 2**; Graphs 1, 2).

**Figure 4:**
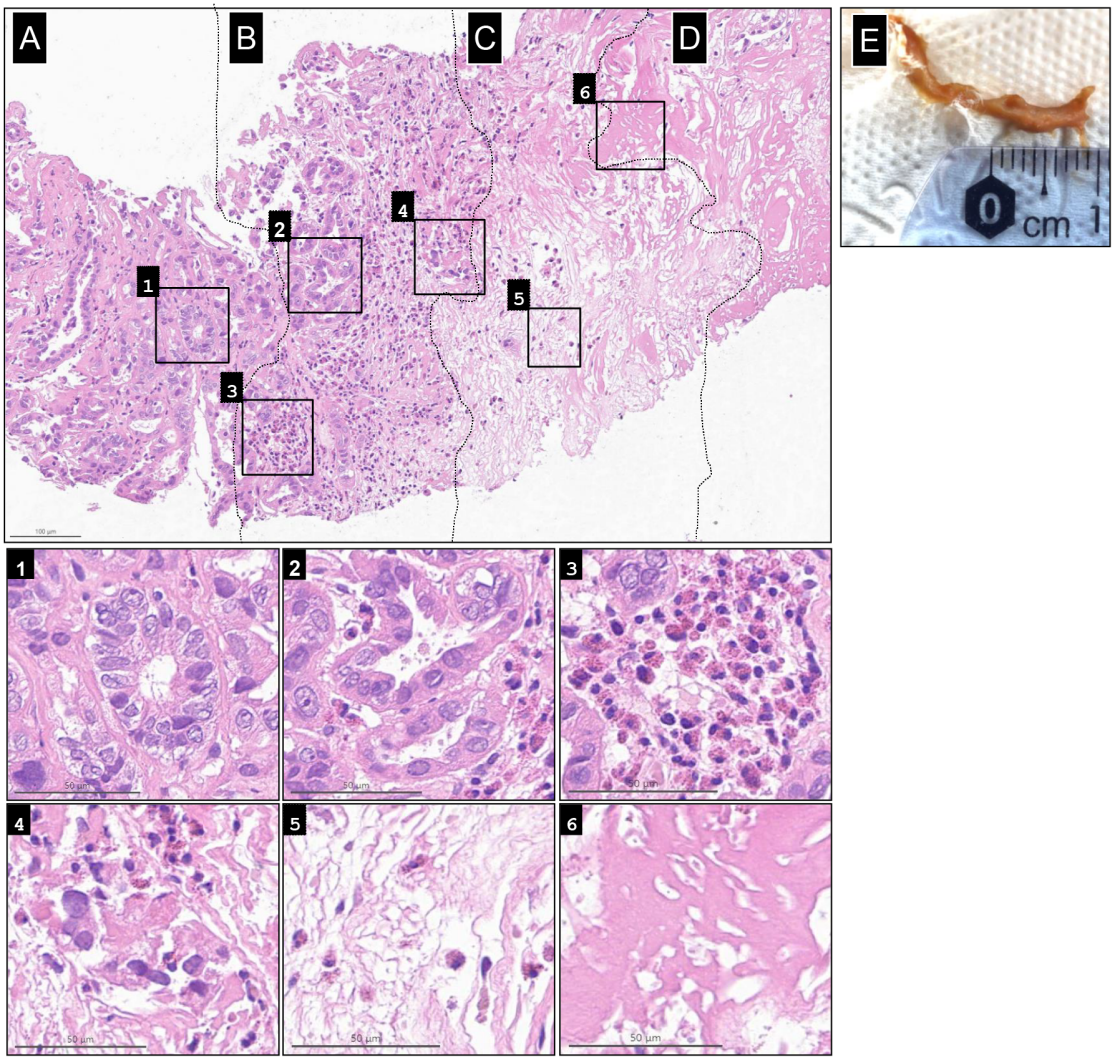
Spectrum of histological changes in renal cell carcinoma under oncolytic virus immunotherapy. Shown from left to right is the full spectrum progression of histological changes, from **(A)** baseline renal cell carcinoma (RCC) tumor, to **(B)** active immunotherapy, **(C)** necrotic tumor tissue, and **(D)** collagen-rich, scar-like tissue induced by oncolytic virus therapy. Additional histological changes from each stage are shown in the six magnified inlets. The vital tumor tissue at the periphery shows only mild to no cytopathic changes in the renal tubuli (1). These changes increased in intensity within the “cancer immune front”, as deformation of tumor glands and cells became more pronounced, accompanied by invasion of tumor glands by eosinophilic granulocytes (EG), increased cytoplasmic vacuolization, and some pycnotic nuclear changes (2). Massive recruitment of EG (3) led to the destruction and fragmentation of tumor glands; EGs remained present. (4). This progressed to complete tissue necrosis with associated edema, with EGs still evident (5). Finally, the central condensed necrotic core developed into collagen-rich, scar-like connective tissue (6). **(E)** Gross specimen of the necrotic tumor cast in urine.

Biopsies from the IT-OV injection sites consistently correlated with the clinical and radiological findings. Pathological examination of a specimen taken in January 2022 (OV 18) revealed the full spectrum of histological changes of RCC under OV therapy. An ongoing immune response was evident near vital tumor tissue, with pronounced cytopathic changes and infiltration of eosinophilic granulocytes leading to tumor necrosis, which subsequently evolved into collagen-rich, scar-like connective tissue (**Figure 4**). Remarkably, microscopic observations not only fully supported the CT and PET-CT results but also revealed the presence of persistent vital tumor tissue for almost 2 years after treatment initiation. However, no vital tumor tissue was detected in biopsies obtained after 2 years.

Blood samples were routinely collected to investigate potential correlations between biomarkers and clinical response to OV immunotherapy. Given the lack of established biomarkers for RCC monitoring (19), levels of C-reactive protein (CRP) and tumor M2-pyruvate kinase (tu-M2-PK) were measured over time. CRP, an inflammatory marker with a normal value of < 5.0 mg/L, increased from a normal pretreatment level of 2.1 to 71.7 mg/L within 16 days of OV 1, and then gradually normalized over the course of treatment. Tu-M2-PK, with a normal value of < 15 U/mL, was mildly elevated before treatment (29 U/mL), peaked at 1,993 U/mL after OV 1, and gradually normalized during subsequent OV treatments, correlating with clinical and radiological findings.

Overall, the treatments were reasonably well tolerated. Within 10 h of the first treatment, the patient developed a fever of up to 39.5°C lasting several hours, followed by cold-like symptoms for 12 days and intermittent bouts of night sweats. Mild hematuria was also observed, along with the shedding of necrotic tumor tissue cast in the urine (**Figure 4F**), which were confirmed by pathological examination to be necrotic, nonmalignant tissue. Similar adverse reactions occurred after subsequent treatment sessions but were milder and of shorter duration. All AEs were transient, reversible, and did not require hospitalization. During OVs 1–6, the patient experienced a 10% loss in his body weight, coinciding with radiological evidence of tumor shrinkage.

During the course of treatment, the patient experienced two episodes of subclinical and asymptomatic intermittent pyelonephritis, detected on imaging and successfully treated with oral antibiotics. Immune-related AEs—including symmetrical maculopapular skin rash predominantly on the upper back and neck, elbow joint pain, asymptomatic small pleural effusion, and mild chest pain—were similar to those reported with immune checkpoint inhibitors (20). These AEs were initially pronounced but later became mild and persisted throughout the treatment duration. After six treatment sessions, the patient returned to baseline weight and regained sufficient strength to perform daily physical activities, and maintained a normal QoL.

As of 5 years after diagnosis (February 2025), the patient remains in CR with fully preserved functional status and QoL.

## 3 Discussion

Standard treatments for RCC-IV—such as nephrectomy, immunotherapy, and other modalities—are associated with significant AEs that negatively impact QoL, while the overall prognosis remains poor. In contrast, our patient’s IT-OV treatments resulted in gradual tumor shrinkage over 3 years and were well tolerated, with preserved QoL, self-limiting AEs, and no need for surgery or hospitalization. Regular radiological monitoring demonstrated characteristic immune-related changes followed by a 90% reduction in the initial mass, with the residual tissue histologically confirmed as scar tissue. Overall, repeated imaging confirmed a complete response, with no evidence of disease (NED) 5 years after diagnosis. To the best of our knowledge, this patient presents the first reported case of successful clinical application of IT-OV therapy for RCC-IV, with detailed imaging and pathological findings documenting NED. Conclusions and suggestions for future clinical studies on the management of advanced RCC are discussed below.

1. OVs have demonstrated effective activity against RCC cells (21–23), and combinations of different OVs have enhanced tumor eradication in animal studies (11). However, to the best of our knowledge, no clinical data confirmed by radiological–pathological correlation have been reported to support curative approaches using OV therapy for RCC treatment (16, 24). The choice and dosages of the OVT regimen were based on published data demonstrating the efficacy of all three viruses in advanced cancer (25–27). The optimal selection of OVs, their combinations, and treatment regimens for RCC should be further investigated in preclinical and clinical studies.
2. IT injection significantly enhances the therapeutic effects of OV therapy compared to systemic administration (15), and repeated administrations are generally well tolerated. QoL was maintained, and all OV treatments were delivered in an outpatient (ambulatory) setting. The use of a nephrostomy with the catheter tip positioned directly in the tumor may allows for more convenient and frequent local IT-OV administration, potentially improving QoL further and reducing the need for repeated, labor-intensive interventional radiological procedures.
3. Cross-sectional imaging, combined with metabolic response monitoring and surveillance, is essential for documenting the immune-mediated effects of IT-OV treatment (28) and for assessing the potential to reduce treatment dose and frequency. Such imaging also provides valuable information into immune activation at the precursor level in typical sites of hematopoiesis. The observed transient AEs correlated with distinct radiological findings and reflect the pronounced systemic immunological response to IT-OV treatment.
4. Repeat biopsies, readily obtainable during US- or CT-guided IT-OV injections, were collected over the course of IT-OV treatment to assess tissue status. The pathological analyses found an increasing proportion of necrotic tissue, suggestive of incremental tumor oncolysis. Tumor fragments found in the urine samples also confirmed necrotic tissue. The radiological–pathological correlation might be useful for monitoring the effectiveness of IT-OV, including assessment of the extent of immune cell infiltration into the necrotic tumor tissue (19). While the success of our experimental IT-OV treatment was demonstrated for a patient with papillary RCC, representing a lower-grade pathology (29), it should be considered for other renal tumors including high-grade clear-cell RCC.
5. Laboratory evaluation of CTC counts, immune function, and viral load may serve as useful tools for monitoring the therapeutic success of IT-OV. Although Tu-M2-PK levels have been reported to be elevated in metastatic RCC (30), they are not yet established as a routine tumor marker (31). Nevertheless, the tu-M2-PK and CRP levels measured over the treatment course for our patient correlated with the therapeutic course.
6. IT-OVs were administered in an intensified, locally injected regimen to optimize therapeutic efficacy while minimizing systemic AEs. The IT-OV injections produced a strong local oncolytic effect accompanied by the intended systemic immune activation, resulting in immunological reactions characteristic of immunotherapy. These AEs were transient and mild and did not require hospitalization.
7. Prior cytotoxic chemotherapy can impair immune function and reduce the efficacy of immunotherapy in cancer (32). In this case, virotherapy was initiated immediately after diagnosis, before any chemotherapy was administered, which likely preserved immune competence and enabled a robust immune response without neutropenia. The observed flare-up in SUV signals in the vertebral bodies, sternum, and long bones was suggestive of the activation of precursor hematopoietic cells induced by IT-OV, which correlated with prolonged high body temperature after IT-OV 1. Furthermore, the gradual shrinkage of the renal mass parallel to the increased uptake in the various LN stations underscores the importance of a functional immune system. The stronger immune response of the LN closer to the treated kidney confirms effective immunotherapy at the tumor-related sites (**Figures 1**, **2**).
8. Despite the Despite NED in the most recent biopsies and imaging studies, continued preventive IT-OV administration at extended intervals—combined with regular radiological and pathological evaluations—may be essential to ensure full eradication of residual tumor cells and to establish long-term cure. Whether the remaining scar/tumor tissue should be surgically resected remains a matter of debate, as the residual tumor may serve as an optimal target for ongoing IT-OV injections to induce local inflammation for optimal immunotherapy (33).

The radiological and pathological remission with NED observed in this patient suggests that IT-injected OV combinations can improve the clinical course of patients with RCC-IV. Based on the successful experimental outcome, further clinical investigation of this innovative approach is encouraged for patients with advanced RCC and potentially for those with other solid tumors. Future investigations of immune markers, tumor markers, viral load, and radiological data, along with regular biopsies might provide important monitoring tools to understand and improve the therapeutic potential of OVT.

## 4 Patient perspective

At first, I was a little unsure when the doctor told me I would be the first patient with renal cell carcinoma he would treat using this approach. At the same time, we did not see any real alternatives, as my treating urologist did not consider any of the conventional options to be promising. After all, the tumor measured over 10 cm. Psychologically, I felt motivated because I knew the viruses would not cause the kind of “collateral damage” that is often inevitable with chemotherapy. I also wanted to preserve my kidney.

The first virus injection was both highly effective and physically stressful, as I developed a high fever and was bedridden for 2 weeks. I lost a significant amount of weight in just a few weeks and could truly feel my body struggle. Chills, feverish episodes, loss of appetite, and extreme fatigue marked the beginning of my therapy. However, since the initial imaging already showed that my immune system was responding vigorously, I remained highly motivated. The first PET scan after the virus injection was very promising.

Overall, I was very happy to have completed my therapy entirely on an outpatient basis. I was diagnosed just before the coronavirus pandemic began (14 February 2020), and if I had been treated as an inpatient, I would have been completely isolated. Instead, I was at home, and my family cared for me with love and support. This, of course, was a tremendous psychological help. Physically, I feel like my old self again. I recently completed a very strenuous move and felt strong throughout. Looking back, I can say that I have not noticed any long-term effects from the treatment. My blood pressure is still slightly elevated, and I continue to take medication for it. However, I stopped taking all other medications a long time ago.

## Data Availability

The datasets presented in this article are not readily available because patient privacy-related concerns. Requests to access the datasets should be directed to the corresponding author.

## In memoriam

The authors would like to commemorate the late Arno Thaller, who was involved in the patient’s initial treatment.
